# Legal Parameters of Practice in Psychiatry

**DOI:** 10.1192/bjo.2022.352

**Published:** 2022-06-20

**Authors:** Christina Barmpagianni, Patrick Cremin, Antonio Fialho, Afaf Qazi

**Affiliations:** Surrey and Borders Partnership NHS Foundation Trust, Epsom, United Kingdom

## Abstract

**Aims:**

The amended Mental Health Act (MHA) of 2007 gave Psychiatrists the right to detain, assess and treat individuals with mental health disorders, not only with a view to offer medical treatment but also to ensure their safety and that of the public, by containing them. This meant that patients diagnosed with disorders such as Antisocial Personality (APD), previously un-detainable under the MHA of 1983, would no longer be considered untreatable and could be sectioned, if appropriate. The idea was then generated, that Psychiatrists would now assume the role of custodians of potentially dangerous people and raised the concern that all persons with APD would be perceived as dangerous and find themselves at a dynamic risk of being sectioned under the revised MHA. The balance between the role of Psychiatrists as medical professionals versus this new, unpopular role as figures of public order was and still is, debatable.

**Methods:**

We present the case of a patient with a background of Depression and Post-traumatic Stress Disorder with aggressive features, who during a consultation revealed a powerful homicidal urge and fantasies directed to an individual he believed had wronged him. The patient had access to the individual and had attempted to confront him. He had no forensic history, nor had he expressed criminal intent before. This triggered a safeguarding response, the consensus being that advice should be sought from the Forensics team, not only to protect the potential victim but also the potential perpetrator from the consequences of a criminal act.

**Results:**

Considering the lack of police involvement, plans, or weapons; the separation between patient and potential victim; and the patient's distress associated with the disclosure of the homicidal fantasies, the level of risk was deemed to not merit disclosure. Closer risk assessment with ongoing psychological and pharmacological interventions created a therapeutic alliance which allowed for open communication with regards to the dynamic nature of the risk and the potential for any further disclosure.

**Conclusion:**

Within the definition of Duty of Care lie responsibilities beyond the strictly medical role of clinicians. Not unlike the duty to inform the DVLA about a patient's fitness to drive, breaking confidentiality for the purposes of patient or public safety is not a power that makes Psychiatrists figures of Authority, but a responsibility that is part of their role. At the same time, we should bear in mind that the license to disclose is also a license not to disclose.

